# Cataract Surgery in Patients with Carotid Cavernous Fistula

**DOI:** 10.4103/0974-9233.58412

**Published:** 2009

**Authors:** Jitendra Jethani

**Affiliations:** Dr. Thakorbhai V Patel Eye Institute, Vadodara, India

Sir,

We read with interest the article by Chaudhary *etal.*,^1^ on ophthalmological implications of carotid cavernous fistula. The article has been compiled nicely and has included almost all the information regarding the ophthalmological implication and effects of carotid cavernous fistula.

However, one of the most common surgeries in ophthalmology is cataract surgery. Cataract surgery in carotid cavernous fistula has been reported and it has important issues regarding the surgery.

Slochower *etal.*,^2^ first reported the problems regarding the surgery of a patient with carotid cavernous fistula. This has been subsequently reported by Hagan *etal* . and Jethani *etal.*^3^,^4^

If cataract surgery is being planned in patients with carotid cavernous fistula, risks such as bleeding during the retrobulbar block, bleeding during conjunctival incision and from the iris vessels and, finally, suprachoroidal hemorrhage from increased choroidal pressure secondary to the venous pressure should be taken into account.^4^

It is important to understand that the pressure in the anterior chamber must be maintained; otherwise, there would be blood in the anterior chamber which would make the surgery difficult.^2^,^3^

We believe that this should be included in the review article so that the readers can be careful while handling/operating such a patient for cataract.
